# CT-Mapped Fusion Patterns After 360° Transforaminal Lumbar Interbody Fusion (TLIF) for Degenerative Spondylolisthesis: High Facet and Interbody Fusion Rates With Limited Clinical Correlates

**DOI:** 10.7759/cureus.98535

**Published:** 2025-12-05

**Authors:** Carla García-Ramos, Diego-Alberto Nuñez-Arreola, Diana-Laura Hernandez Moctezuma, Alejandro Antonio Reyes-Sanchez, Armando Alpizar-Aguirre, Baron Zarate-Kalfopulos, Irving O Estevez-Garcia, Ernesto Roldan-Valadez

**Affiliations:** 1 Spinal Surgery, Instituto Nacional de Rehabilitación "Luis Guillermo Ibarra Ibarra", Mexico City, MEX; 2 Research, Instituto Nacional de Rehabilitación "Luis Guillermo Ibarra Ibarra", Mexico City, MEX; 3 Radiology, I.M. Sechenov First Moscow State Medical University, Moscow, RUS

**Keywords:** clinical outcomes, degenerative spondylolisthesis, fusion success rates, lumbar spine fusion surgery, oswestry disability index (odi), postoperative complications, quality of life, roland-morris questionnaire, transforaminal lumbar interbody fusion (tlif)

## Abstract

Introduction: Degenerative spondylolisthesis is a common cause of low back pain and disability. We evaluated fusion rates across predefined lumbar regions and their association with clinical outcomes after transforaminal lumbar interbody fusion (TLIF). We aimed to compare CT-confirmed fusion across predefined anatomical zones and to assess baseline-to-12-month changes in Oswestry Disability Index (ODI) and Roland-Morris Disability Questionnaire (RMDQ).

Methods: We retrospectively analyzed 88 patients who underwent 360° TLIF at the Mexican National Rehabilitation Institute (January 2017-May 2022). Fusion at seven anatomical zones was assessed on a 12-month follow-up CT. Patient-reported outcomes at 12 months included the ODI and the RMDQ.

Results: Operations most commonly involved L4-L5 (38; 43.1%), followed by L4-L5+L5-S1 (31; 35.3%), L3-L4+L4-L5 (12; 13.7%), L5-S1 (5; 5.9%), and L2-L3+L3-L4+L4-L5 (2; 2.0%). Fusion success was high, particularly for interbody fusion (79; 89.6%, p < 0.0001) and facet joint fusion. Complications were uncommon (8; 9.1%): pedicle fracture (2; 2.3%), durotomy (1; 1.1%), dural injury (1; 1.1%), transient nerve alteration on neuromonitoring (2; 2.3%), L4 spinous-process fracture (1; 1.1%), and screwdriver breakage with retained fragment (1; 1.1%). Fusion location/number did not correlate with postoperative ODI (p = 0.7124) or RMDQ (p = 0.4255). Several fusion types were more often successful in female patients (p < 0.01).

Discussion: TLIF achieved high CT-confirmed fusion-especially interbody and facet-yet the anatomical distribution/number of fused zones did not influence 12-month disability (ODI/RMDQ). Results likely reflect surgical technique and patient optimization rather than fusion location. Interpretation should be cautious given the retrospective single-center design, 12-month follow-up, lack of bone-quality data, and exploratory multiple comparisons.

## Introduction

The term spondylolisthesis, first introduced by Killian in 1853, denotes anterior displacement of one vertebra relative to the one below; posterior displacement of the superior vertebra is termed retrolisthesis [1]. Instability arises within the paravertebral joint complex: vertebral body, intervertebral disc, and facet joints [2]. Degenerative spondylolisthesis is the most frequent form in individuals over 50 years of age and is a leading cause of lower back pain and disability worldwide [3]. Its prevalence in the general population is estimated at 3-5% and appears to affect all genders and ethnic groups [4]. Low back pain is the leading global cause of years lived with disability; in 2020, approximately 619 million people were affected, with projections approaching 843 million by 2050, and occupational exposures, smoking, and high BMI accounting for a substantial share of attributable burden [5]. In older adults, prevalence rises after age 50 and progression is faster in women [6].

The clinical spectrum ranges from mild discomfort to severe neurologic compromise (paresthesia, lower-limb pain, saddle anesthesia, and sphincter dysfunction). Failure of conservative management frequently necessitates surgery [7]. Beyond physical symptoms, degenerative spondylolisthesis imposes considerable psychosocial and economic costs, limiting work capacity and daily functioning [8]. These features underscore the need for durable treatments that restore spinal stability [9] and improve quality of life [10].

Transforaminal lumbar interbody fusion (TLIF) is a widely accepted option for surgical stabilization. The technique removes the intervertebral disc and places a bone graft within the disc space to achieve interbody fusion. Compared with posterior lumbar interbody fusion (PLIF) and anterior lumbar interbody fusion (ALIF), TLIF offers a less invasive corridor, preserves the interspinous ligament and contralateral lamina, and reduces the risk of dural tears and nerve-root injury [7,11-13]. Clinical studies report high fusion rates with TLIF-approaching 90% in some cohorts, with concomitant improvements in validated outcome measures such as the Oswestry Disability Index (ODI), and Roland-Morris Disability Questionnaire (RMDQ) [11,12]. Nonetheless, TLIF is technically demanding and has a learning curve that emphasizes the importance of surgical experience. Contemporary TLIF, including minimally invasive approaches, commonly achieves >90% one-year fusion rates [10].

Despite these successes, few studies have examined whether the anatomical distribution of fusion influences recovery. Evidence remains scarce on how fusion in specific lumbar zones-interbody, facet, posteromedial, and intertransverse-relates to changes in functional scales, and little is known about these relationships in Mexican populations [12]. Some reports suggest fusion location may not materially affect recovery [7,11-13], whereas emerging CT-based work indicates that consolidation may differ among posterior and interbody regions and that radiographic fusion does not always track with patient-reported benefit [14-16]. Differentiating anatomical sites of fusion is clinically relevant because graft placement strategy and expectations for stability may differ when facet/posterolateral fusion predominates versus interbody fusion; standardized CT mapping at 12 months addresses variability in prior reports.

We conducted a retrospective cohort study of patients who underwent 360° TLIF at a tertiary referral center (2017-2022). Our objectives were to quantify and compare computed-tomography-confirmed fusion at 12 months across four predefined zones (interbody space, facet joint, posteromedial gutter, and intertransverse region), to evaluate change in disability from baseline to 12 months using the ODI and the RMDQ, and to examine whether the anatomical location-or the total number-of successful fusions correlated with postoperative disability (ODI, RMDQ). We hypothesized that fusion rates would differ by zone and that any correlation with disability would be small.

## Materials and methods

This was a retrospective study involving adult patients who underwent single-position, posterior-only 360° TLIF with pedicle-screw instrumentation for degenerative spinal disease at the Instituto Nacional de Rehabilitación “Luis Guillermo Ibarra Ibarra”, Mexico, between January 2017 and May 2022. The protocol was approved by the Institutional Review Board (62/23 AE-2025-1) and conducted in accordance with national and international standards of Instituto Nacional de Rehabilitación "Luis Guillermo Ibarra Ibarra" (Mexican Official Standard NOM-012-SSA3-2012) and was conducted as per the Declaration of Helsinki, 1975, revised 2013.

Study population

Eligible participants were 18-75 years old with single- or multilevel degenerative lumbar conditions and radiological signs of instability. Diagnostic criteria included one or more of the following: facet or endplate osteophytosis, decreased disc height, scarring/fissures of the facet capsule, annulus, or ligamentum flavum, disc herniation, facet arthropathy, and/or vacuum phenomenon. Only patients with ≥12 months of postoperative follow-up were included. In total, 88 patients met the criteria, all treated with posterior instrumented fusion using local autologous bone graft. Sex (male/female) was abstracted from the medical record; gender identity was not collected.

Surgical team and setting 

All procedures were performed by the same spine surgery team of board-certified orthopedic surgeons (two attending spine surgeons performed all procedures, each with >10 years of independent practice). Fellows in spine surgery assisted under direct supervision.

Surgical technique

All patients underwent 360° TLIF. The procedure comprised discectomy, interbody cage placement, and pedicle screw fixation. Autologous bone graft harvested during decompression was placed in the interbody space and targeted posterior fusion zones to optimize arthrodesis. 

Implants and Approach

Interbody cages were Adonis polyetheretherketone (PEEK) TLIF cages (HumanTech Spine GmbH, Steinenbronn, Germany), typically 7-8 mm in height, used with the Adonis pedicle screw-rod system (HumanTech). A single-position 360° TLIF was performed through a single incision using a posterior-only approach. Instrumentation consisted of a titanium polyaxial pedicle-screw/rod system.

Graft Material and Preparation

Local autograft only was used (no bone morphogenetic protein (BMP) or other expanders). Morselized bone harvested from facets, lamina, and, when available, vertebral elements was packed within the cage and into the anterior disc space after careful endplate preparation.

Posterior Fusion Technique

After neural decompression, bilateral facetectomy was performed as indicated. Posterior elements and transverse processes were aggressively decorticated, and morselized autograft was placed in the facet joints and intertransverse gutters to promote posterolateral fusion.

Perioperative Management and Rehabilitation

All patients followed a standardized pathway: early mobilization on postoperative day 1 and brace use for comfort only. A structured physiotherapy program (core/hip strengthening, gait training, and progressive conditioning) began at week 2 and continued per tolerance. Outpatient reviews were scheduled at six weeks, three months, and 12 months, and lumbar CT at 12 months of adjudicated fusion. Analgesia included a COX-2-selective non-steroidal anti-inflammatory drug (NSAID) course for 10 days postoperatively; no systemic corticosteroids were prescribed. Smoking cessation was reinforced perioperatively for all patients.

Radiological assessment of fusion

**Figure 1:**
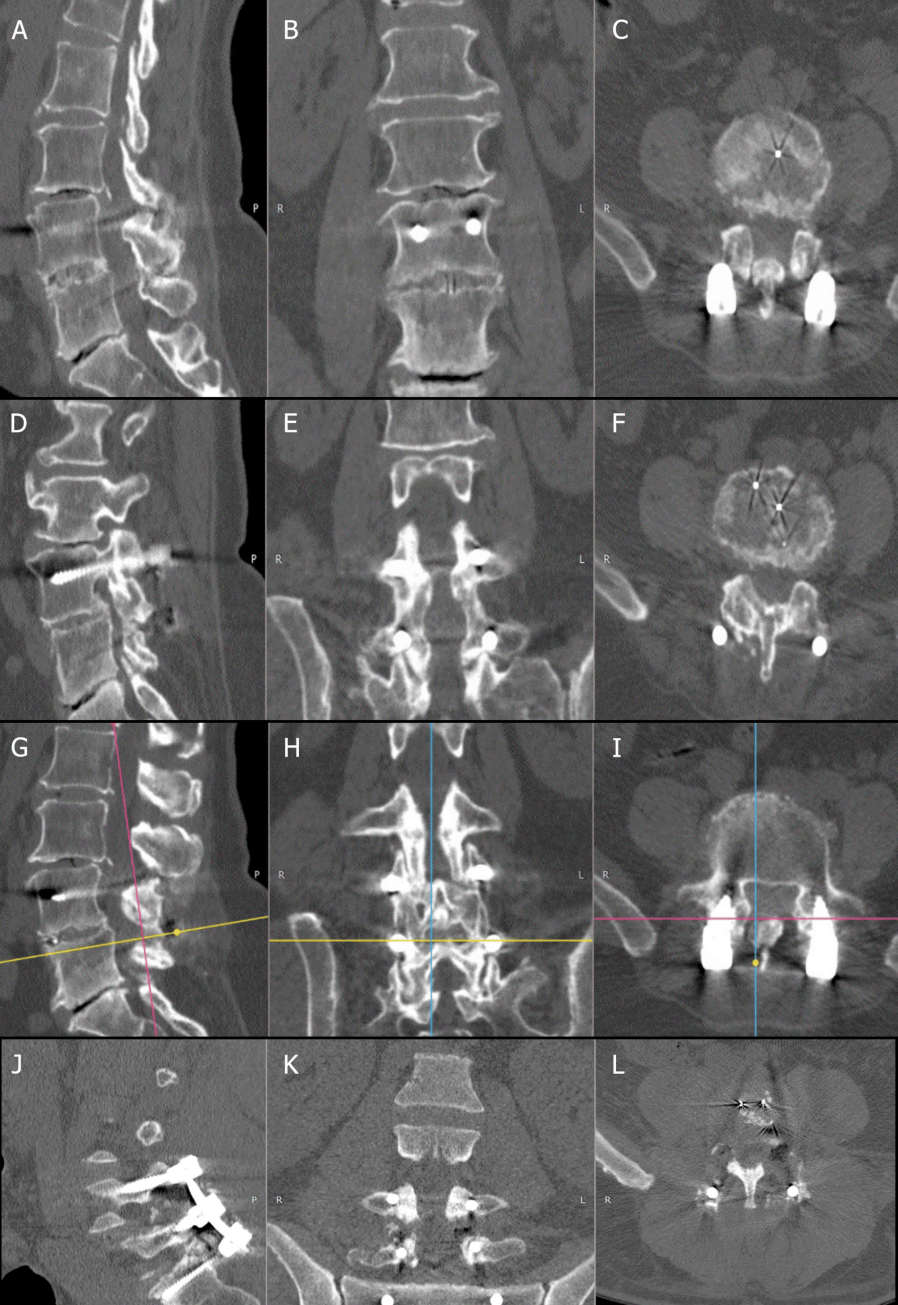
Multiplanar CT adjudication of lumbar fusion across four anatomical sites from the study cohort. Examples shown on bone-window reformats (slice thickness ≤1.25 mm; bone kernel) in the three orthogonal planes. A–C, Intertransverse (posterolateral) space: sagittal (A), coronal (B), axial (C); fusion = trabecular bridging across the decorticated intertransverse gutters and adjacent transverse processes. D–F, Interbody space: sagittal (D), coronal (E), axial (F); fusion = continuous trabecular bone traversing the adjacent endplates through/around the cage without an intervening cleft. G–I, Facet joint space: sagittal (G), coronal (H), axial (I); fusion = osseous bridging/obliteration of the facet joint after decortication. J–L, Posteromedial space: sagittal (J), coronal (K), axial (L); fusion = bridging along the lamina/spinous processes within the medial gutters. Operational rule: fusion required continuous trabecular bridging on ≥2 consecutive slices in the same plane or confirmation on orthogonal planes within the same zone.

The primary objective was to compare fusion rates across predefined zones: facet joint fusion (FJF), interbody fusion (IBF), posteromedial fusion (PMF), and Intertransverse (posterolateral) fusion (PLF). At 12 months postoperatively, CT scans were obtained to determine fusion status. Multidetector CT with thin-slice reconstructions (≤ 1.5 mm) using a bone kernel was acquired; sagittal and coronal MPRs were generated for zone-by-zone assessment. CT studies were read independently by two experienced spine surgeons and a board-certified musculoskeletal radiologist; discrepancies were adjudicated by consensus. Fusion was defined as trabecular bone bridging visible on at least two consecutive images on the same plane (axial/sagittal/coronal) or across orthogonal planes within the same zone. Seven anatomical sites were evaluated: right/left PLF, IBF, right/left FJF, and right/left PMF. Representative reconstructions are shown in Figure 1. On dynamic radiographs, translation >4 mm on flexion-extension was considered instability.

Clinical outcome measures and study tools

Functional outcomes were collected at baseline and 12 months using the ODI v2.1a [17,18] and the RMDQ [19]. Instruments were administered and scored according to their manuals. Permission to use the ODI was acquired from Mapi Research Trust, Lyon, France (https://eprovide.mapi-trust.org). For this study, we used the official Mexican-Spanish ODI v2.1a [20]. Formal email authorization to use the Mexican-Spanish ODI v2.1a was granted by the corresponding author of the validation study [20].

ODI v2.1a consists of 10 sections scored 0-5; raw totals (0-50) were multiplied by two to yield a 0-100% index (higher = worse). Conventional severity bands were used for descriptive context: 0-20 minimal, 21-40 moderate, 41-60 severe, 61-80 crippled, 81-100 bed-bound. The RMDQ contains 24 yes/no items (range 0-24; higher = greater limitation).

Statistical analysis

Data are summarized as mean ± SD for approximately normal distributions and median (interquartile range (IQR)) otherwise. Between-group comparisons (e.g., female vs male) for continuous variables used an independent-samples t-test when normal and the Mann-Whitney U test when non-normal (U reported). Within-subject pre/post comparisons used paired t-tests or the Wilcoxon signed-rank test as appropriate. Complete-case analysis was used for paired pre- vs post-operative comparisons (only patients with both timepoints were analyzed for each instrument); missing data were not imputed. Categorical variables were compared with Pearson’s chi-square; when any expected cell count was < 5, Fisher’s exact test was applied (χ² or Fisher p reported). Correlations between fusion metrics and outcomes were measured using Spearman’s ρ. Correlation analyses across anatomical zones were pre-specified but treated as exploratory given multiple comparisons. Distributional assumptions were checked with Kolmogorov-Smirnov and Shapiro-Wilk tests. Because several comparisons were performed across anatomical zones, findings are considered exploratory, and p-values were not adjusted for multiplicity; effect sizes and directional consistency are reported to contextualize significance testing. All tests were two-sided with α = 0.05. Analyses were performed with IBM SPSS Statistics for Windows, version 27.0.1.0 (Released 2020; IBM Corp., Armonk, New York, United States). Microsoft Excel v16.33 (Microsoft Corporation, Redmond, Washington, United States) was used for data management and table preparation.

## Results

Demographic characteristics

A total of 88 patients were included (mean age 63.4 ± 11.7 years). Age did not differ by sex (female patients 62.67 ± 11.0 years vs. male patients 65.00 ± 13.4 years; p = 0.559). Female patients had lower weight (66.61 ± 6.8 kg vs. 73.13 ± 6.9 kg; p < 0.005) and shorter height (1.57 ± 0.10 m vs. 1.66 ± 0.10 m; p = 0.0001); BMI was similar (p = 0.520) (Table 1). Smoking prevalence was 35-40% with no significant sex difference.

**Table 1:** Demographic characteristics of the study cohort by sex (N=88) Female–male comparisons used the two-sided Mann–Whitney U test; U statistics are shown.

Characteristics	Total, mean±SD	Female (n=61), mean±SD	Male (n=27), mean±SD	U	p-value
Age (years)	63.40 ± 11.7	62.67 ± 11.0	65.00 ± 13.4	759	0.559
Weight (kg)	68.65 ± 7.4	66.61 ± 6.8	73.13 ± 6.9	513	<0.005
Height (m)	1.60 ± 0.10	1.57 ± 0.10	1.66 ± 0.10	394	0.0001
BMI (kg/m²)	26.81 ± 2.1	26.67 ± 2.1	27.09 ± 2.1	752	0.520

Surgical levels of posterior instrumented fusion surgeries (PIFS)

Sex-specific patterns were observed across levels. All three-level fusions (L2-L3, L3-L4, L4-L5) occurred in women (5/5; p = 0.0085). Single-level L4-L5 procedures were more common in women (76.47%) than men (23.53%; p = 0.0251), and all single-level L5-S1 cases occurred in women (16/16; p < 0.0001). Overall, women accounted for 68.75% of surgeries versus 31.25% for men (p = 0.0002) (Table 2).

**Table 2:** Distribution of single- and multilevel PIFS by sex. Between-sex comparisons used Pearson’s χ²; Fisher’s exact test was applied when any expected cell count was < 5 or a zero cell occurred (χ² column shows “— (Fisher’s exact)” and the p-value column reports the Fisher p). χ² statistics are shown with degrees of freedom (df) and rounded to two decimals. The Total row reports the omnibus χ² test across the five-level categories (df = 4). PIFS: posterior instrumented fusion surgery

Surgical level	Female, n (%)	Male, n (%)	χ² (df)	p-value
L2–L3, L3–L4, L4–L5 (3 levels)	5 (100.0)	0 (0.0)	— (Fisher's exact)	0.0085
L3–L4, L4–L5 (2 levels)	6 (33.3)	12 (66.7)	2.04 (1)	0.153
L4–L5, L5–S1 (2 levels)	26 (65.0)	14 (35.0)	3.57 (1)	0.059
L4–L5 (1 level)	13 (76.5)	4 (23.5)	5.02 (1)	0.0251
L5–S1 (1 level)	16 (100.0)	0 (0.0)	— (Fisher's exact)	<0.0001
Total	66 (68.8)	30 (31.3)	22.00 (4)	0.0002

Fusion success by anatomical site and sex

Female patients exhibited higher success proportions for several fusion types: intersomatic fusion (72.09% vs. 27.91%; p = 0.0032), right facet fusion (71.43% vs. 28.57%; p = 0.010), and left facet fusion (75.86% vs. 24.14%; p = 0.0043). PMF also favored the female sex (right 80.00% vs. 20.00%, p = 0.0055; left 87.50% vs. 12.50%, p = 0.0015). Intertransverse fusion showed no significant sex difference (right p = 0.123; left p = 0.0572) (Table 3).

**Table 3:** Comparison of fusion success rates by anatomical site and sex. Values are number (percent) of successful fusions. Between-sex comparisons used Pearson’s χ²; degrees of freedom (df) are shown in parentheses, and χ² values are rounded to two decimals. The Total row reports the omnibus χ² across the six anatomical sites (df = 6); because p < 0.0001, the statistic is presented as a lower bound (χ² ≥ 27.86).

Type of fusion	Female, n (%)	Male, n (%)	χ² (df)	p-value
Intersomatic fusion	31 (72.1)	12 (27.9)	8.69 (1)	0.0032
Right facet fusion	25 (71.4)	10 (28.6)	6.63 (1)	0.010
Left facet fusion	22 (75.9)	7 (24.1)	8.15 (1)	0.0043
Right posteromedial fusion	16 (80.0)	4 (20.0)	7.71 (1)	0.0055
Left posteromedial fusion	14 (87.5)	2 (12.5)	10.08 (1)	0.0015
Right intertransverse fusion	14 (66.7)	7 (33.3)	2.38 (1)	0.123
Left intertransverse fusion	16 (69.6)	7 (30.4)	3.62 (1)	0.0572
Total	133 (73.1)	49 (26.9)	≥ 27.86 (6)	< 0.0001

Comparison with international literature

Relative to Kim et al. [21], our cohort achieved higher intersomatic fusion success (89.58% vs. 55.6%; p < 0.0001), whereas right posteromedial fusion was lower (41.67% vs. 73.4%; p = 0.0011). Other site-specific comparisons also differed significantly, as detailed in Table 4.

**Table 4:** Comparison of fusion success in PIFS with Kim et al. (CT at 12 months) Kim et al.'s denominators [21]: PLF (n = 94), PLF+IBF (n = 63). Between-group comparisons used Pearson’s χ² (df = 1 for each 2×2 row); when any expected cell count was < 5 or a zero cell occurred, Fisher’s exact test was applied (the χ² column shows “—” and the p-value reflects Fisher’s exact). Where p < 0.0001, the χ² statistic is presented as a lower bound (e.g., χ² ≥ 15.14). PIFS: posterior instrumented fusion surgery; PLF: posterolateral fusion; IBF: interbody fusion

Type of fusion	Rate of current study, n (%)	Rate of Kim et al. [21], n (%)	χ² (df)	p-value
Intersomatic (IBF)	43 (89.58%)	35 (55.6%) (of 63 PLF+IBF)	≥ 15.14 (1)	<0.0001
Right facet fusion	35 (72.92%)	N/A	—	N/A
Left facet fusion	29 (60.42%)	N/A	—	N/A
Right posteromedial fusion	20 (41.67%)	69 (73.4%) (of 94 PLF)	10.65 (1)	0.0011
Left posteromedial fusion	16 (33.33%)	69 (73.4%) (of 94 PLF)	≥ 15.14 (1)	<0.0001
Right intertransverse fusion	21 (43.75%)	33 (35.1%) (of 94 PLF)	≥ 15.14 (1)	<0.0001
Left intertransverse fusion	23 (47.92%)	33 (35.1%) (of 94 PLF)	6.93 (1)	0.0085

Correlation between fusion success and cage subsidence

**Figure 2:**
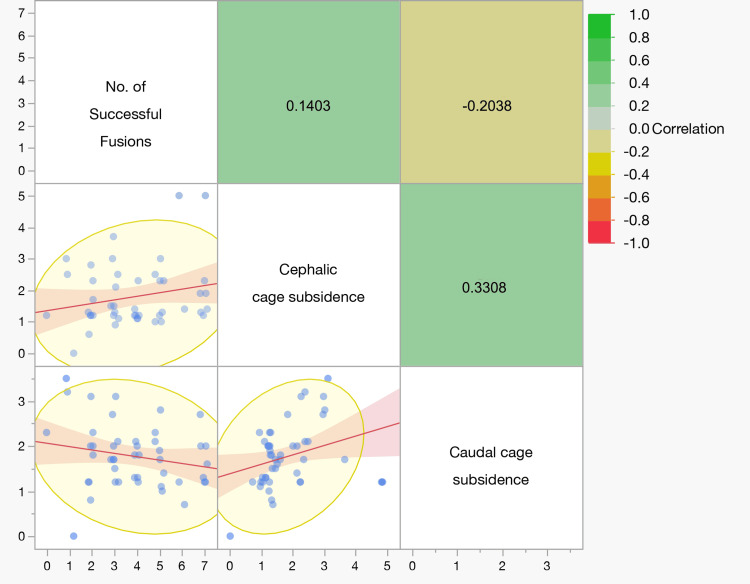
Correlation matrix between number of successful fusions and cage subsidence. Upper-right cells show Spearman’s ρ (color‐coded by magnitude/direction: green = positive, yellow/orange = negative; values rounded to two decimals). Lower-left cells display scatter plots with fitted regression lines, 95% confidence bands (shaded), and 95% data ellipses. A positive correlation was observed between cephalic and caudal subsidence (ρ ≈ 0.33, p = 0.023), whereas correlations between the number of successful fusions and either subsidence measure were small and not statistically significant.

Cephalic and caudal cage subsidence were positively correlated (Spearman’s ρ = 0.3308; p = 0.0231). The number of successful fusions did not correlate with cephalic (ρ = 0.1403; p = 0.3523) or caudal (ρ = −0.2038; p = 0.1743) subsidence (Table 5, Figure 2).

**Table 5:** Correlation between fusion success rate and cage subsidence after TLIF. Spearman’s rank correlation coefficients (ρ) and two-sided p-values assess associations between fusion-rate success and cage subsidence at the cephalic and caudal endplates, and the correlation between subsidence at both levels. Positive ρ denotes a direct correlation; negative ρ denotes an inverse correlation. Statistical significance was set at α = 0.05. TLIF: transforaminal lumbar interbody fusion

Variable 1	Variable 2	Spearman ρ	p-value
Cephalic cage subsidence	Fusion rate success	0.1403	0.3523
Caudal cage subsidence	Fusion rate success	-0.2038	0.1743
Cephalic cage subsidence	Caudal cage subsidence	0.3308	0.0231

Clinical outcome measures

From baseline to 12 months, median ODI improved from 28.0 (IQR, 21.5-30.0) to 20.0 (IQR, 18.0-24.0); Wilcoxon Z = −3.66; p = 0.0002; r = 0.56. Median RMDQ increased from 18.0 (IQR, 15.5-18.2) to 20.0 (IQR, 18.0-22.0); Z = 4.43; p < 0.0001; r = 0.70. Taken together, these findings indicate improvement on ODI but worsening on RMDQ, underscoring instrument-dependent differences and the need for cautious clinical interpretation at 12 months (Table 6).

**Table 6:** Patient-reported outcomes at baseline and 12 months after TLIF Paired differences failed Shapiro–Wilk normality; therefore, all comparisons used the Wilcoxon signed-rank test, reported as Z with two-sided p-values and effect size r = |Z|/√N (N = number of non-zero pairs). Higher scores indicate worse disability for ODI (0–100%) and RMDQ (0–24). Oswestry Disability Index contact information and permission to use: Mapi Research Trust, Lyon, France, https://eprovide.mapi-trust.org TLIF: transforaminal lumbar interbody fusion; ODI: Oswestry Disability Index; RMDQ: Roland–Morris Disability Questionnaire; IQR: interquartile range

Scale	Baseline, median (IQR)	12 months, median (IQR)	Test	Statistic (Z)	p-value	Effect (r)
ODI (%)	28.0 (21.5–30.0)	20.0 (18.0–24.0)	Wilcoxon	−3.66	0.0002	0.56
RMDQ (0–24)	18.0 (15.5–18.2)	20.0 (18.0–22.0)	Wilcoxon	4.43	<0.0001	0.70

Correlation between fusion success and clinical scales

At 12 months, the number of successful fusions did not correlate with ODI total (ρ = −0.0552; p = 0.7124), or RMDQ (ρ= 0.1190; p = 0.4255) (Table 7).

**Table 7:** Correlations between number of successful fusions and postoperative patient-reported outcomes at 12 months Spearman’s ρ with two-sided p-values quantifies associations between fusion success and postoperative scores. Higher values reflect worse disability for ODI and RMDQ. Positive ρ indicates that higher postoperative scores are associated with more successful fusions; negative ρ indicates the opposite. Oswestry Disability Index contact information and permission to use: Mapi Research Trust, Lyon, France, https://eprovide.mapi-trust.org ODI: Oswestry Disability Index; RMDQ: Roland–Morris Disability Questionnaire

Variable 1	Variable 2	Spearman ρ	p-value
Number of successful fusions	ODI total	−0.0552	0.7124
Number of successful fusions	RMDQ	0.1190	0.4255

Surgical levels and associated complications

Complications were uncommon (8/88; 9.1%) and distributed across levels: pedicle fracture/screw pull-out (n = 2; 2.3%), durotomy (n = 1; 1.1%), dural injury (n = 1; 1.1%), transient anterior tibial nerve alteration on neuromonitoring (n = 2; 2.3%), L4 spinous-process fracture (n = 1; 1.1%), and screwdriver breakage with retained fragment (n = 1; 1.1%). The highest level-specific proportion occurred in L4-L5+L5-S1 procedures (4/31; 12.9%), followed by L3-L4+L4-L5 (1/12; 8.3%) and single-level L4-L5 (3/38; 7.9%); none occurred at L5-S1 (0/5) or L2-L3+L3-L4+L4-L5 (0/2) (Table 8).

**Table 8:** Postoperative complications by surgical level (N=88) Complications recorded within 12 months of TLIF, reported by level in n (%) using level-specific denominators: L4–L5 (38), L4–L5+L5–S1 (31), L3–L4+L4–L5 (12), L5–S1 (5), L2–L3+L3–L4+L4–L5 (2). Percentages in body cells are calculated per level (e.g., two complications at L4–L5+L5–S1 → 2/31 = 6.5%). The rightmost column expresses totals as a proportion of the entire cohort (N = 88). “Any complication” indicates ≥ 1 event at that level. TLIF: transforaminal lumbar interbody fusion

Complication	L4–L5	L4–L5 + L5–S1	L3–L4 + L4–L5	L5–S1	L2–L3 + L3–L4 + L4–L5	Frequency (Percentage)
Pedicle fracture (L5 pedicle/screw pull-out)	0 (0.0%)	2 (6.5%)	0 (0.0%)	0 (0.0%)	0 (0.0%)	2 (2.3%)
Durotomy	1 (2.6%)	0 (0.0%)	0 (0.0%)	0 (0.0%)	0 (0.0%)	1 (1.1%)
Dural injury	1 (2.6%)	0 (0.0%)	0 (0.0%)	0 (0.0%)	0 (0.0%)	1 (1.1%)
Nerve alteration (neuromonitoring)	0 (0.0%)	2 (6.5%)	0 (0.0%)	0 (0.0%)	0 (0.0%)	2 (2.3%)
L4 spinous-process fracture	0 (0.0%)	0 (0.0%)	1 (8.3%)	0 (0.0%)	0 (0.0%)	1 (1.1%)
Screwdriver breakage/retained fragment	1 (2.6%)	0 (0.0%)	0 (0.0%)	0 (0.0%)	0 (0.0%)	1 (1.1%)
Any complication	3 (7.9%)	4 (12.9%)	1 (8.3%)	0 (0.0%)	0 (0.0%)	8 (9.1%)

## Discussion

Principal findings

The primary technical goal of lumbar fusion is to establish a solid osseous union between adjacent vertebrae, either via PLF across the intertransverse processes or IBF linking adjacent endplates [2,22]. In this cohort undergoing 360° TLIF for degenerative spondylolisthesis, fusion most consistently consolidated in the intersomatic and facet regions. Fusion success was more frequent in women than in men, a difference that may partly reflect greater adherence to immediate, intermediate, and late postoperative follow-up among female patients.

Anatomical fusion patterns and imaging considerations

Although radiographic confirmation of fusion has been associated with better outcomes, the correlation is inconsistently reported [21]. CT-based mapping studies indicate that posterolateral and facet arthrodesis can equal or exceed interbody consolidation in some series, aligning with the strong facet fusion observed here. Use of multiplanar CT improves detection over plain radiographs when adjudicating union and localizing bridging trabeculae [14]. 

Radiographic fusion vs patient-reported outcomes 

In this cohort, the number and location of successful fusions were not associated with 12-month disability scores (Table 7; all |ρ| ≤ 0.12; p > 0.42). Notably, ODI improved whereas RMDQ worsened, indicating discordant trajectories across instruments at the same time point, reinforcing prior observations that “solid fusion” on imaging does not necessarily translate into superior patient-reported outcomes after lumbar fusion [15,16]. Potential mediators include baseline disability, the adequacy of neural decompression, segmental/global alignment, bone health, and rehabilitation intensity. Possible contributors include the RMDQ’s activity-item sensitivity, rehabilitation variability, and residual pain generators not captured by fusion distribution. Accordingly, our correlation results are exploratory and support a conservative inference: at 12 months, fusion burden/distribution was not a detectable driver of disability in this dataset. This message should inform counseling (see Table 7).

Clinical counseling and decision-making

In practical terms, the absence of correlation between fusion burden/distribution and 12-month disability (Table 7; Figure 3) suggests that perioperative counseling should emphasize decompression quality, global/sagittal alignment, bone health, and rehabilitation adherence as the primary drivers of recovery rather than the exact anatomic site(s) of bony consolidation. Achieving solid arthrodesis remains necessary for long-term mechanical stability, yet short-term patient-reported outcomes are likely more sensitive to relief of neural compression and restoration of biomechanics. Accordingly, surgical planning should prioritize meticulous decompression and alignment targets, with fusion site selection tailored to exposure, stability needs, and patient risk profile rather than expectations of PRO gains at 12 months.

Comparison with international literature

Compared with the multicenter series by Kim et al. [21], intersomatic fusion in our cohort was higher, possibly reflecting technical refinements such as placement of locally harvested bone into the interbody space in conjunction with the implant. Conversely, right posteromedial fusion was lower than reported internationally. These differences may relate to surgeon handedness, asymmetric neural or dural compression, or intraoperative positioning that affects access and graft placement on one side (Table 4).

Sex differences and potential confounders

The higher consolidation rates observed in female patients across multiple zones may reflect unmeasured differences in bone quality, biomechanics, or care pathways. Although smoking prevalence was similar by sex and all patients received a short, standardized course of COX-2-selective NSAIDs without corticosteroids, bone mineral density and rehabilitation intensity were not systematically captured. These factors, together with technical nuances (e.g., graft packing, access asymmetry), could contribute to the sex disparity and should be evaluated prospectively.

Clinical implications

High consolidation in intersomatic and facet regions supports TLIF as a robust stabilizing procedure when performed by experienced teams, consistent with prior reports linking solid fusion to improved function in degenerative spondylolisthesis [8]. Nonetheless, our data suggest that quality-of-life gains after TLIF may be driven more by decompression and pain relief than by the specific anatomical site of osseous union.

Cage subsidence and risk factors

Cephalic and caudal cage subsidence were positively related, suggesting a shared biomechanical substrate. Known risk factors-age, BMI, and bone quality-mirror those reported by Liu and Li [23]. Although we did not detect an outcome penalty at 12 months, reviews and meta-analyses identify older age, endplate injury, and low bone quality as consistent risk factors for subsidence after TLIF; osteoporosis is linked to higher risks of cage migration and screw loosening [24,25]. These observations support meticulous endplate preparation and preoperative bone-health optimization (Table 5, Figure 2).

Correlation with clinical scales

No significant associations were found between the anatomical location of fusion and improvements on the ODI or RMDQ. Thus, while achieving solid arthrodesis remains important, the precise fusion site may not directly determine functional recovery. Prior work similarly indicates that successful fusion, regardless of site, can yield meaningful clinical benefit [26]. 

Complications associated with surgical levels

Complications were infrequent and distributed across levels, with events including pedicle fracture/screw pull-out, durotomy, dural injury, L4 spinous-process fracture, transient anterior tibial nerve alteration on neuromonitoring, and screwdriver breakage with a retained fragment. Given the mechanical demands and exposure challenges at L4-L5, careful preoperative planning, meticulous technique, and neuromonitoring remain advisable (Table 8).

Strengths and limitations

This study leveraged thin-slice CT with predefined anatomical zones and blinded review, providing a granular map of arthrodesis after 360° TLIF. Nevertheless, several limitations warrant caution. First, this was a retrospective, single-center design, and a modest cohort distributed across five surgical level combinations, reducing power for subgroup analyses; the retrospective design with potential selection bias and rehabilitation variability may influence patient-reported outcomes independently of fusion distribution. Second, bone quality was not measured with dual-energy X-ray absorptiometry (DEXA)/bone mineral density (BMD), a key confounder for both fusion and subsidence and a potential contributor to the observed sex differences. Third, follow-up was limited to 12 months, adequate for early fusion but insufficient for durability, adjacent-segment disease, or late clinical change. Fourth, we conducted multiple hypothesis tests across anatomical zones; p-values should be interpreted as exploratory, and consistency of effects is emphasized over isolated significances. Fifth, RMDQ scores worsened despite ODI improvement. This discordance may reflect instrument differences (activity-based sensitivity of RMDQ), rehabilitation variability, or residual pain generators unrelated to the fusion bed; however, it underscores that our null correlations between fusion distribution and patient-reported outcomes at 12 months should be interpreted conservatively and validated prospectively. The ODI-RMDQ divergence further tempers clinical inference and supports validation in prospective, longer-term cohorts with standardized rehabilitation and bone-quality assessment.

Future research

Prospective, multicenter studies should test whether technical refinements, biological augmentation, and standardized rehabilitation protocols improve fusion success and recovery. Taken together, the evidence suggests that patient optimization, technical execution, and global alignment may influence outcomes more than the exact anatomical distribution of fusion, offering a plausible explanation for the weak correlations observed here [15,27]. Despite standardized perioperative care and similar smoking prevalence by sex, residual confounding from unmeasured patient factors or technical nuances cannot be excluded.

## Conclusions

In degenerative spondylolisthesis treated with 360° TLIF, fusion consolidated most reliably in the interbody (intersomatic) and facet regions on 12-month CT. Across the cohort, the anatomical distribution/number of fused zones did not relate to 12-month patient-reported disability (ODI, RMDQ), suggesting that surgical execution and patient optimization (e.g., bone health, alignment, rehabilitation) are more consequential for recovery than the specific fusion site.

Complete, stable arthrodesis remains essential for durable symptom control and may influence longer-term phenomena such as adjacent-segment degeneration. Future studies should use prospective designs with extended follow-up and standardized CT criteria to define how modifiable factors-implant selection, graft strategy, and peri-operative optimization-shape both radiographic success and quality of life.
